# Ice Flavor–Related Discussions on Twitter: Content Analysis

**DOI:** 10.2196/41785

**Published:** 2022-11-30

**Authors:** Artur Galimov, Julia Vassey, Ellen Galstyan, Jennifer B Unger, Matthew G Kirkpatrick, Jon-Patrick Allem

**Affiliations:** 1 Department of Population and Public Health Sciences Keck School of Medicine of University of Southern California Los Angeles, CA United States

**Keywords:** electronic cigarettes, Twitter, social media, ice flavors, tobacco policy, public health, infodemiology, FDA, tobacco, smoking, vaping, e-cigarette, public

## Abstract

**Background:**

The US Food and Drug Administration (FDA) recently restricted characterizing flavors in tobacco products. As a result, ice hybrid–flavored e-cigarettes, which combine a cooling flavor with fruit or other flavors (eg, banana ice), emerged on the market. Like menthol, ice-flavored e-cigarettes produce a cooling sensory experience. It is unclear if ice hybrid–flavored e-cigarettes should be considered characterizing flavors or menthol, limiting regulatory action. Monitoring the public’s conversations about ice-flavored e-cigarettes on Twitter may help inform the tobacco control community about these products and contribute to the US FDA policy targets in the future.

**Objective:**

This study documented the themes pertaining to vaping and ice flavor–related conversations on Twitter. Our goal was to identify key conversation trends and ascertain users’ recent experiences with ice-flavored e-cigarette products.

**Methods:**

Posts containing vaping-related (eg, “vape,” “ecig,” “e-juice,” or “e-cigarette”) and ice-related (ie, “Ice,” “Cool,” “Frost,” and “Arctic”) terms were collected from Twitter’s streaming application programming interface from January 1 to July 21, 2021. After removing retweets, a random sample of posts (N=2001) was selected, with 590 posts included in the content analysis. Themes were developed through an inductive approach. Theme co-occurrence was also examined.

**Results:**

Many of the 590 posts were marked as (or consisted of) marketing material (n=306, 51.9%), contained positive personal testimonials (n=180, 30.5%), and mentioned disposable pods (n=117, 19.8%). Other themes had relatively low prevalence in the sample: neutral personal testimonials (n=45, 7.6%), cannabidiol products (n=41, 7%), negative personal testimonials (n=41, 7%), “official” flavor description (n=37, 6.3%), ice-flavored JUUL (n=19, 3.2%), information seeking (n=14, 2.4%), and comparison to combustible tobacco (n=10, 1.7%). The most common co-occurring themes in a single tweet were related to marketing and disposable pods (n=73, 12.4%).

**Conclusions:**

Our findings offer insight into the public’s experience with and understanding of ice-flavored e-cigarette products. Ice-flavored e-cigarette products are actively marketed on Twitter, and the messages about them are positive. Public health education campaigns on the harms of flavored e-cigarettes may help to reduce positive social norms about ice-flavored products. Future studies should evaluate the relationship between exposure to personal testimonials of ice-flavored vaping products and curiosity, harm perceptions, and experimentation with these products among priority populations.

## Introduction

The use of e-cigarettes, or vaping, among adolescents and young adults is a public health concern [1,2]. One factor contributing to e-cigarette use among adolescents and young adults is product diversity and the availability of appealing flavors [3,4]. Use of flavored e-cigarettes like fruit, sweet, mint, and menthol are the most widely used flavors among adolescents and young adults [4-6]. In February 2020, to counteract e-cigarette use among adolescents and young adults, the US Food and Drug Administration (FDA) restricted the manufacture, distribution, marketing, and sale of prefilled cartridge-based e-cigarettes (eg, JUUL) in flavors other than menthol and tobacco [7]. Additionally in July 2020, the US FDA issued warning letters to 10 prominent e-cigarette manufactures, including Cool Clouds Distribution, the parent company of the popular brand Puff Bar, requesting that they remove their flavored disposable e-cigarette products because they lacked the required premarket authorization [8,9]. In the context of these regulations, ice hybrid–flavored products were introduced into the market [3,7]. Ice hybrid–flavored products combine a cooling flavor with fruit, dessert, or other characterizing flavor (eg, blueberry ice or melon ice), which may allow e-cigarette companies to circumvent regulatory action [10,11]. Recent evidence suggests that the use of ice-flavored e-cigarettes among young adults may be common and positively associated with combustible tobacco use [10,12].

Ice hybrid–flavored products may contain menthol. Menthol is a flavor additive that produces pleasant cooling sensations and analgesic effects in the throat and mouth, which reduces the harshness of combustible smoke and nicotine’s irritating effects on the airways [11,12]. Research from human and animal studies indicates that menthol increases the intensity of how cigarettes are smoked (eg, deeper inhalation) and facilitates smoking initiation and nicotine intake [11,13,14]. In addition, studies have demonstrated that menthol additives in e-liquids mask the harshness and irritation associated with vaping nicotine and increases the liking of vaping products [12,15,16]. Many ice-flavored e-cigarette products contain synthetic coolants (eg, menthone, eucalyptol, peppermint oil, and WS-3) that produce comparable or stronger menthollike cooling and counterirritant effects [11,12,14]. In fact, even low levels of these cooling additives, which may not have been classified as a characterizing flavor, could increase appeal, promote deeper aerosol inhalation, and encourage more frequent e-cigarette use [11,12,15]. Thus, ice flavors may not fit into existing flavor profile categories, such as characterizing flavors (eg, fruit) or menthol, which may circumvent current regulatory efforts [10].

One approach that may be helpful in informing regulatory efforts is the tracking and analyzing of real-world conversations and depictions of novel tobacco products on Twitter [17]. Monitoring tobacco-related content on Twitter can provide valuable insights into the public’s beliefs, attitudes, and experiences surrounding new tobacco products [18-20]. Engagement (eg, seeing, posting, liking, and sharing) with tobacco-related content and posting about tobacco products on Twitter, Facebook, and other social media is associated with an increased risk of tobacco use [21,22]. While Twitter users have been shown to be vulnerable to the effects of positive messages about vaping, to the best of our knowledge, prior research has not assessed ice flavor discussions with Twitter data [23].

This study used Twitter data to document the themes in posts pertaining to ice flavors. Examining and monitoring the public’s conversations about ice-flavored e-cigarettes may help inform the tobacco control community about these products and uncover trends, knowledge, and attitudes toward e-cigarette flavors. Findings from this study may contribute to the US FDA policy targets and tobacco control campaigns in the future.

## Methods

### Overview

Twitter posts containing both vaping-related (eg, “vape,” “ecig,” “e-juice,” “e-cigarette,” or “JUUL”; see Multimedia Appendix 1 for the complete list of keywords) and “Ice,” “Cool,” “Frost,” or “Arctic” terms were collected from January 1 to July 21, 2021, from Twitter’s streaming application programming interface. A total of 976,347 posts containing these terms were identified. Similar to previous studies, after excluding all retweets, we sampled out a random subset of 2001 posts for a content analysis [18,20]. Two coders (AG and EG) worked together with the last author (JPA) to become familiar with the data, created a codebook, and coded tweets into themes using an inductive approach. The unit of analysis was the text of the tweet. The purpose of the approach was to summarize the raw text-based data into a summary format and report the underlying themes evident in the data. Saturation was determined to be reached with 10 themes.

Vaping-related posts that contained the terms “Ice,” “Cool,” “Frost,” or “Arctic” but were determined to be unrelated to our research objectives were identified through manual content analysis and removed. For instance, irrelevant posts contained the following phrases: “cool vape trick” and “vaping is cool.” After excluding irrelevant and non-English posts, we were left with 590 (29.5%) tweets that were included in the content analysis.

The codebook consists of the following themes: *personal testimonials* (which were further divided into the following five subcategories: *positive sentiment*, *negative* *sentiment*, *neutral sentiment*, *information seeking*, and *comparison to combustible tobacco*), *marketing*, *official (promotional) flavor description*, *disposable pod devices*, *JUUL*, *cannabidiol* (*CBD*) *products*, and o*ther*. A tweet could be classified into multiple themes. To establish interrater reliability, the same subsample of posts (590 of the original 2001 posts) were double coded, with percent agreement ranging from 99.2% to 100% and Cohen κ ranging from 0.90 to 1.00. JPA served as an arbitrator resolving discrepancies between the coders.

**Figure 1:**
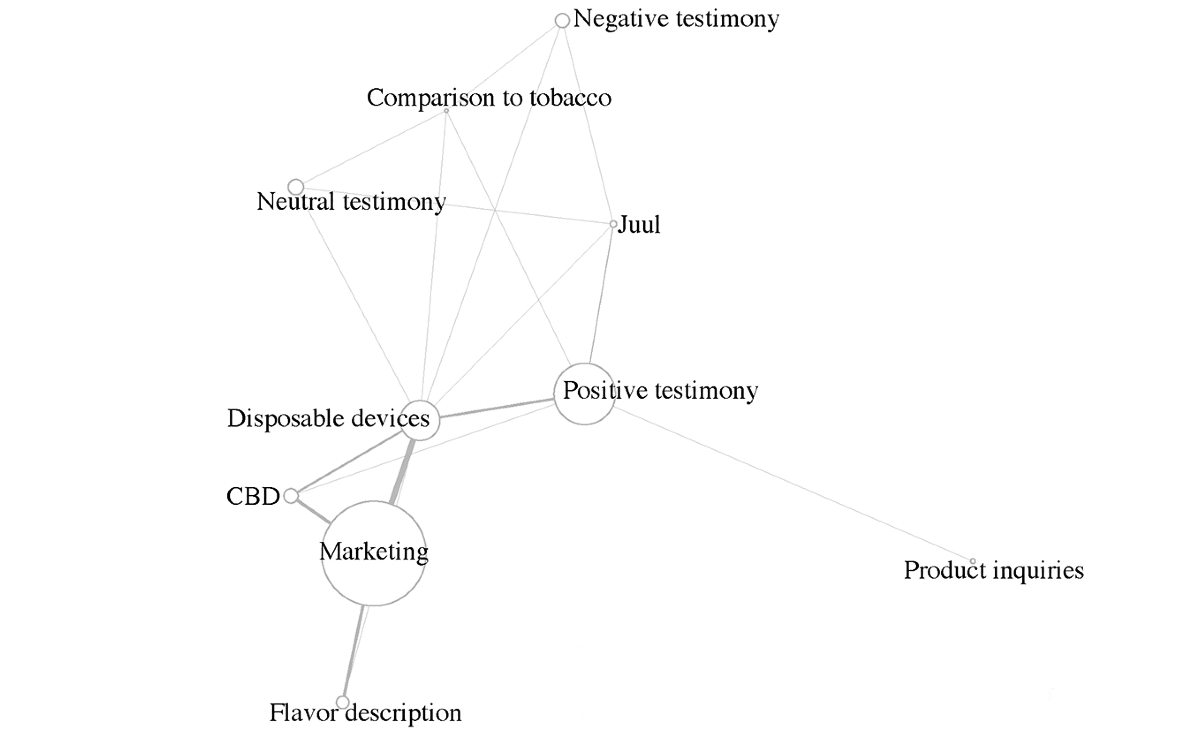
Co-occurrences of themes in corpus tweets. The size of the circles represents the frequency of the theme occurrences. The proximity of the circles and the size of the lines represent the frequency of the theme pairwise co-occurrences. CBD: cannabidiol.

Descriptive analyses were conducted first to show the prevalence of each theme. Further, pairwise co-occurrence analyses were used to evaluate connections across themes, since tweets often included more than one theme. R base package (version 4.0.2; R Foundation for Statistical Computing) was used to construct the co-occurrence matrix, and the igraph package (version 1.2.6) was used to visualize the results. Since the theme related to *personal testimonials* consisted of *positive*, *negative*, and *neutral testimonies*, Figure 1 does not include the overarching *personal testimonials* theme, and only includes its components: *positive*, *negative*, and *neutral testimonies*. The *other* category was also excluded since it had few co-occurrences with other themes.

All Twitter posts in this data set were publicly available and anonymized, and all analyses adhered to the terms and conditions, terms of use, and privacy policies of Twitter. To further protect privacy, posts exemplifying themes were paraphrased; no tweets are reported verbatim.

### Ethical Approval

The protocol was approved by the university’s institutional review board (protocol HS-18-00697).

## Results

Descriptive characteristics of the 590 tweets from this corpus and example paraphrased posts are presented in Table 1. Overall, the most prominent themes were *marketing* (n=306, 51.9%) of ice-flavored products and *personal testimonials* (n=277, 47%). Mentions of using ice-flavored *disposable pod devices* (n=117, 19.8%) was also a common theme. Other themes had relatively low prevalence in the sample: “ice-flavored” *CBD products* (n=41, 7%), *official flavor description* (n=37, 6.3%), and ice-flavored *JUUL* (n=19, 3.2%). *Positive sentiment* (n=180, 30.5%) was the most prevalent theme among personal testimonials, while the other testimonial themes were less common: *neutral sentiment* (n=45, 7.6%), *negative sentiment* (n=41, 7%), *information seeking* (n=14, 2.4%), and *comparison to combustible tobacco* (n=10, 1.7%).

The most commonly co-occurring themes in a single tweet were related to the most prominent themes in the data set: *marketing*, *positive sentiment*, and *disposable pod devices*. The marketing of ice-flavored disposable pod devices was mentioned in 73 (12.4%) of the 590 tweets; the marketing of ice-flavored CBD products was mentioned in 40 (6.8%) of the tweets; the marketing of ice-flavored products and official flavor descriptions was mentioned in 37 (6.3%) of the tweets. Positive testimonies about disposable devices were observed in 31 (5.3%) of the tweets, and conversations about disposable devices containing CBD were observed in 26 (4.4%) of the tweets. Pairwise co-occurrences of the remaining themes were found in 2% of the tweets (see Figure 1, where the thickness of the lines represents the frequency of pairwise co-occurrences and the size of the circles represents the frequency of theme occurrences).

**Table 1:** Definition for each theme, descriptive statistics, and selected paraphrased twitter posts.

Theme	Posts (n=590), n (%)	Definition	Paraphrased post
Positive sentiment testimonials	180 (30.5)	Posts containing a positive experience with (eg, tastes, smells great) or favorable opinion about (eg, love, like, my favorite) “ice-flavored” tobacco products, including e-liquids	“Lychee Ice is the best e-cigarette flavor”
Negative sentiment testimonials	41 (7.0)	Posts containing a negative experience with (eg, tastes, smells horrible, bad) or negative opinion about (eg, hate, dislike) “ice-flavored” tobacco products, including e-liquids	“Recently bought a banana ice vape and it tastes horrible...”
Neutral sentiment testimonials	45 (7.6)	Posts containing a neutral opinion about “ice-flavored” tobacco products, including e-liquids (eg, if no valence is determinable or deemed neutral)	“This tangerine-apple ice e-cigarette tastes like 7-up”
Information seeking	14 (2.4)	Posts where a consumer or potential consumer asks for information on or opinions about “ice-flavored” tobacco products, including e-liquids	“Do you guys know if a mango ice e-liquid tastes good?”
Comparison to combustible tobacco	10 (1.7)	Posts containing mentions of or comparisons between “ice-flavored” products, including e-liquids and combustible tobacco products	“...I am not planning to switch from my menthol cigarettes to vaping, but I will definitely buy one of these lychee ice flavored disposables...”
Marketing	306 (51.9)	Posts promoting/selling/marketing an “ice-flavored” e-liquids or devices	“Guava ice e-liquid is now available at https://xxx...”
Disposable pod devices	117 (19.8)	Mentions of “ice-flavored” disposable pod devices (ie, Puff Bar)	“Banana Ice Puff Bar is worth every penny...”
JUUL	19 (3.2)	Mentions of “ice-flavored” JUUL	“@xxx Ice flavored JUUL is the best JUUL”
CBD^a^ products	41 (7.0)	Mentions of “ice-flavored” CBD vaping products	“New CBD Blackcurrant Ice Disposable vapes are now in stock at https://xxx...”
Official flavor description	37 (6.3)	Posts containing an official (ie, “media,” promotional, marketing) description of “ice-flavored” e-liquids or products	“...The sweet juice of Fuji apples combined with a hint of nectarines & strawberry to create a well rounded fruity vape experience with a cool refreshing finish...”
Other	6 (1.0)	Any other posts that contain ice flavor–related themes and do not fit into any category listed in the codebook	“Use of ice-flavored e-cigarettes may be associated with nicotine dependence. See https://xxx...”

^a^CBD: cannabidiol.

## Discussion

### Principal Findings

This study provides a summary of public Twitter posts collected over the course of a 7-month period, which includes mentions of both vaping-related and ice flavor–related terms. Posts in our corpus were related to ice-flavored e-cigarette product marketing, personal testimonials, and ice-flavored disposable pod devices. Theme co-occurrence in a single post was examined.

Marketing was a common theme in this study, while marketing and disposable pod devices represented a common theme co-occurrence in a single post. These findings are consistent with recent studies suggesting that ice-flavored products are often promoted in various pod-style cartridge-based disposable (eg, Puff Bar) and refillable (eg, PUFF Krush, PHIX) e-cigarette products [11]. These disposable and refillable pod-style products are among the fastest-growing segments of the e-cigarette market [3,9,10]. To reduce the appeal of e-cigarette products among youth, the US FDA took regulatory action (February 2020) to remove prefilled cartridge-based e-cigarettes in flavors other than menthol and tobacco, and issued warning letters to 10 e-cigarette companies instructing them to stop selling flavored disposable e-cigarettes by July 2020 [7,8]. Although some companies (eg, JUUL) voluntarily removed all flavors except menthol and tobacco, other companies (including those producing JUUL-compatible pods) continued to manufacture them. Given that ice hybrid–flavored products may contain both cooling and fruity flavors, it is unclear how these flavors fit into current regulatory policies. While the US FDA is moving toward removing menthol from combustible tobacco products, it is critical that regulatory actions consider restricting any constituents that taste/smell like menthol and produce a similar cooling sensory experience (ie, ice flavors) [12,24].

*Personal testimonials* was another common theme in this study, while *positive sentiment* was the most common category among testimonials. These findings are consistent with previous studies showing that Twitter users are exposed to positive messages about vaping and tobacco [25,26]. Positive messages about tobacco on social media may be linked with tobacco product experimentation [21]. Given that the messages about the ice-flavored products on Twitter are positive and that user conversations are not subject to federal policies, public health education campaigns informing the public on the harms of flavored e-cigarettes may be beneficial in countering these messages and reducing positive social norms about these products [21,26]. For instance, it could be possible to target individuals posting positive messages about ice-flavored e-cigarette products and deliver cessation messages directly to these individuals and their social networks. In addition, the US FDA regulations banning tobacco marketing on Twitter may help to limit the promotion of ice flavors to the public.

### Limitations

This study was limited to the analysis of discussions on ice-flavored e-cigarette products and may not pertain to other e-cigarette flavors. In addition, relatively few (n=2001, 0.2%) tweets among all identified ice tweets were analyzed (n=976,347), which limits the generalizability of our results. Moreover, the word “cool” was one of the four terms used to identify ice-flavored e-cigarette posts. “Cool” is a colloquialism and is commonly used to express the enjoyment of someone or something. The inclusion of this search term resulted in a collection of many posts that were unrelated to e-cigarette flavors; nonetheless, we found this limitation unavoidable and worked diligently to analyze pertinent tweets. This study focused on the text of the Twitter posts but did not code website links or accompanying images. Previous studies demonstrated that there is value in examining both text and image. In other words, it is possible that additional themes would have emerged had we coded images. Findings may not generalize to other time periods or other social media platforms. Our findings may not extend to all Twitter users or to the population of the United States.

### Conclusions

Our findings may offer valuable insights into the public’s experience with and understanding of ice-flavored e-cigarette products. In this study, we found that Twitter discussions about ice-flavored e-cigarette products focused on marketing, personal testimonials, and ice-flavored disposable pod devices. Future studies should evaluate the relationship between exposure to personal testimonials of ice-flavored vaping products and curiosity, harm perceptions, and experimentation with these products among priority populations. Public health education campaigns informing the public on the harms of flavored e-cigarettes may be helpful in reducing positive social norms about ice-flavored products, while flavor regulatory actions may include ice flavors to prevent new products from circumventing current regulations. For instance, banning tobacco marketing posts on Twitter may reduce the exposure to ice-flavored e-cigarette product promotions. In addition, social media might be more influential than traditional marketing because participants can be actively engaged in the content they are viewing [21,22]. Close monitoring of ice flavor promotions on social media is needed to ensure that these promotions are not targeted toward minors and e-cigarette nonusers. Twitter has a policy prohibiting paid advertising of tobacco products to appear on its platform [27]. However, promotional posts related to ice-flavored products are still prevalent. Future studies should evaluate whether these promotions are sponsored by e-cigarette manufacturers or e-cigarette product distributors, or generated by general users.

## Appendix

Multimedia Appendix 1 List of keywords.

## Abbreviations

CBD: cannabidiol
FDA: Food and Drug Administration

## Data Availability

The data sets generated or analyzed during this study are available from the corresponding author on reasonable request.

## References

[1] Fuentes AL, Crotty Alexander LE (2022). Beware, vaping e-cigarettes around children is adversely impacting their lung health. Thorax.

[2] Johnston L, Miech R, O'Malley P, Bachman J, Schulenberg J, Patrick M (2021). Monitoring the future national survey results on drug use, 1975-2020: overview, key findings on adolescent drug use. Institute for Social Research, University of Michigan.

[3] Galimov A, Leventhal A, Meza L, Unger JB, Huh J, Baezconde-Garbanati L, Sussman SY (2021). Prevalence of disposable pod use and consumer preference for e-cigarette product characteristics among vape shop customers in Southern California: a cross-sectional study. BMJ Open.

[4] Rostron BL, Cheng Y, Gardner LD, Ambrose BK (2020). Prevalence and reasons for use of flavored cigars and ENDS among US youth and adults: estimates from wave 4 of the PATH study, 2016-2017. Am J Health Behav.

[5] Wang TW, Neff LJ, Park-Lee E, Ren C, Cullen KA, King BA (2020). E-cigarette use among middle and high school students - United States, 2020. MMWR Morb Mortal Wkly Rep.

[6] Zare S, Nemati M, Zheng Y (2018). A systematic review of consumer preference for e-cigarette attributes: flavor, nicotine strength, and type. PLoS One.

[7] (2020). FDA finalizes enforcement policy on unauthorized flavored cartridge-based e-cigarettes that appeal to children, including fruit and mint. US Food and Drug Administration.

[8] (2020). FDA notifies companies, including Puff Bar, to remove flavored disposable e-cigarettes and youth-appealing e-liquids from market for not having required authorization. US Food and Drug Administration.

[9] Cwalina SN, McConnell R, Benowitz NL, Barrington-Trimis JL (2021). Tobacco-free nicotine - new name, same scheme?. N Engl J Med.

[10] Leventhal A, Dai H, Barrington-Trimis J, Sussman S (2021). 'Ice' flavoured e-cigarette use among young adults. Tob Control.

[11] Leventhal AM, Tackett AP, Whitted L, Jordt SE, Jabba SV (2022). Ice flavours and non-menthol synthetic cooling agents in e-cigarette products: a review. Tob Control.

[12] Davis DR, Morean ME, Bold KW, Camenga D, Kong G, Jackson A, Simon P, Krishnan-Sarin S (2021). Cooling e-cigarette flavors and the association with e-cigarette use among a sample of high school students. PLoS One.

[13] Leventhal A, Cho J, Barrington-Trimis J, Pang R, Schiff S, Kirkpatrick M (2020). Sensory attributes of e-cigarette flavours and nicotine as mediators of interproduct differences in appeal among young adults. Tob Control.

[14] Kreslake JM, Wayne GF, Alpert HR, Koh HK, Connolly GN (2008). Tobacco industry control of menthol in cigarettes and targeting of adolescents and young adults. Am J Public Health.

[15] Krishnan-Sarin S, Green BG, Kong G, Cavallo DA, Jatlow P, Gueorguieva R, Buta E, O'Malley SS (2017). Studying the interactive effects of menthol and nicotine among youth: an examination using e-cigarettes. Drug Alcohol Depend.

[16] Rosbrook K, Green BG (2016). Sensory effects of menthol and nicotine in an e-cigarette. Nicotine Tob Res.

[17] Barker JO, Vassey J, Chen-Sankey JC, Allem J, Cruz TB, Unger JB (2021). Categorizing IQOS-related Twitter discussions. Int J Environ Res Public Health.

[18] Allem J, Dharmapuri L, Unger JB, Cruz TB (2018). Characterizing JUUL-related posts on Twitter. Drug Alcohol Depend.

[19] Allem J, Dormanesh A, Majmundar A, Rivera V, Chu M, Unger JB, Cruz TB (2021). Leading topics in Twitter discourse on JUUL and Puff Bar products: content analysis. J Med Internet Res.

[20] Allem J, Escobedo P, Chu K, Soto DW, Cruz TB, Unger JB (2017). Campaigns and counter campaigns: reactions on Twitter to e-cigarette education. Tob Control.

[21] Unger JB, Urman R, Cruz TB, Majmundar A, Barrington-Trimis J, Pentz MA, McConnell R (2018). Talking about tobacco on Twitter is associated with tobacco product use. Prev Med.

[22] Soneji S, Yang J, Knutzen KE, Moran MB, Tan AS, Sargent J, Choi K (2018). Online tobacco marketing and subsequent tobacco use. Pediatrics.

[23] McCausland K, Maycock B, Leaver T, Wolf K, Freeman B, Jancey J (2020). E-cigarette advocates on Twitter: content analysis of vaping-related tweets. JMIR Public Health Surveill.

[24] (2022). FDA proposes rules prohibiting menthol cigarettes and flavored cigars to prevent youth initiation, significantly reduce tobacco-related disease and death. US Food and Drug Administration.

[25] McCausland K, Maycock B, Leaver T, Jancey J (2019). The messages presented in electronic cigarette-related social media promotions and discussion: scoping review. J Med Internet Res.

[26] Allem J, Ramanujam J, Lerman K, Chu K, Boley Cruz T, Unger JB (2017). Identifying sentiment of hookah-related posts on Twitter. JMIR Public Health Surveill.

[27] Tobacco and tobacco accessories. Twitter for Business.

