# Supplementary material for: Ice Flavor–Related Discussions on Twitter: Content Analysis
Source: J Med Internet Res. 2022 Nov 30;24(11):e41785. doi: 10.2196/41785 (PMC9752452; doi:10.2196/41785)
Supplement: Multimedia Appendix 1 [file jmir_v24i11e41785_app1.docx]

**List of Keywords**

**We first identified vaping-related themes using the following keywords:**

1. ecig
2. e-cigs
3. ecigs
4. e-cigarette
5. ecigarette
6. e-cigarettes
7. ecigarettes
8. vape
9. vaper
10. vaping
11. vapes
12. vapers
13. ehookah
14. e-hookah
15. ejuice
16. ejuices
17. e-juice
18. e-juices
19. eliquid
20. eliquids
21. e-liquid
22. e-liquids
23. esmoke
24. e-smoke
25. vapor
26. electronic cigarette
27. juul
28. Phix
29. suorin
30. juuling
31. puffbar
32. Aspire
33. PuffBa
34. IGNITE
35. Flum
36. Lush
37. Posh
38. Hyppe
39. Air Bar
40. Bang XXL
41. Big Bar
42. Big Boy
43. Beas tBar
44. EZZY
45. Fog X Magnum
46. Geek Bar Pro
47. INFZN
48. Pod Twist
49. Tobo
50. Xtra
51. Steam Engine Vape Nunu
52. Steam Engine Vape Monster
53. VAPor LAX
54. Breeze Plus
55. LOY XL
56. Sea XXL
57. Stig
58. JUUL
59. SMOK
60. ASPIRE
61. Suorin
62. PHIX
63. Mi-Pod
64. ion

**Then we searched for 'ice' flavor-related themes within identified tweets using the following keywords:**

1. Ice
2. Cool
3. Frost
4. Arctic
